# Uremic Solutes in Chronic Kidney Disease and Their Role in Progression

**DOI:** 10.1371/journal.pone.0168117

**Published:** 2016-12-29

**Authors:** Jan A. J. G. van den Brand, Henricus A. M. Mutsaers, Arjan D. van Zuilen, Peter J. Blankestijn, Petra H. van den Broek, Frans G. M. Russel, Rosalinde Masereeuw, Jack F. M. Wetzels

**Affiliations:** 1 Department of Nephrology, Radboud Institute of Health Sciences, Radboud University Medical Center, Nijmegen, The Netherlands; 2 Department of Pharmaceutical Technology and Biopharmacy, University of Groningen, Groningen, The Netherlands; 3 Department of Nephrology and Hypertension, University Medical Centre Utrecht, Utrecht, The Netherlands; 4 Department of Pharmacology and Toxicology, Radboud Institute for Molecular Life Sciences, Radboud University Medical Center, Nijmegen, The Netherlands; 5 Division of Pharmacology, Utrecht Institute for Pharmaceutical Sciences, Utrecht, The Netherlands; University of Colorado Denver School of Medicine, UNITED STATES

## Abstract

**Background:**

To date, over 150 possible uremic solutes have been listed, but their role in the progression of CKD is largely unknown. Here, the association between a selected panel of uremic solutes and progression in CKD patients was investigated.

**Methods:**

Patients from the MASTERPLAN study, a randomized controlled trial in CKD patients with a creatinine clearance between 20 and 70 ml/min per 1.73m^2^, were selected based on their rate of eGFR decline during the first five years of follow-up. They were categorized as rapid (decline >5 ml/min per year) or slow progressors. Concentrations of eleven uremic solutes were obtained at baseline and after one year of follow-up. Logistic regression was used to compare the odds for rapid to slow progression by uremic solute concentrations at baseline. Variability in uremic solute levels was assessed using scatter plots, and limits of variability were calculated.

**Results:**

In total, 40 rapidly and 40 slowly progressing patients were included. Uremic solutes were elevated in all patients compared to reference values for healthy persons. The serum levels of uremic solutes were not associated with rapid progression. Moreover, we observed substantial variability in solute levels over time.

**Conclusions:**

Elevated concentrations of uremic solutes measured in this study did not explain differences in rate of eGFR decline in CKD patients, possibly due to lack of power as a result of the small sample size, substantial between patient variability, and variability in solute concentrations over time. The etiology of intra-individual variation in uremic solute levels remains to be elucidated.

## Introduction

Patients with advanced chronic kidney disease (CKD) suffer from a wide array of symptoms, including anemia, bone-mineral disorders, reduced cognitive function and hypertension. These symptoms are thought to be caused, at least partially, by the accumulation of so-called uremic solutes.

Vanholder *et al*. defined uremic solutes as those compounds which accumulate in blood and tissues during the development of end stage kidney disease and which have an impact on biological functions.[1] To date, the European Uremic Solute Workgroup (EUTox) lists over 150 of such retention solutes.[2, 3] Moreover, solutes classified by the EUTox project may be elevated not only in dialysis patients, but in patients with less advanced stages of CKD as well.[4, 5]

Evidence suggests that uremic solutes can induce apoptosis and epithelial-to-mesenchymal transition in the proximal tubule.[6–8] In addition, uremic solutes are substrates but also inhibitors of tubular transport proteins that are responsible for the excretion of drugs, solutes and waste products.[5, 9–16] Furthermore, uremic solutes can alter mitochondrial functioning, resulting in increased superoxide and hydroxyl radical production and increased rates of apoptosis. [17, 18] Uremic solutes have been associated with clinical outcome as well. A recent review suggests a major role for protein-bound uremic solutes in cardiovascular morbidity and mortality.[19] Moreover, sequestration of uremic solutes in the intestine resulted in a slower eGFR decline in CKD patients.[20] However, this is indirect evidence for a role of uremic solutes at best. Therefore, we investigated whether a selection of uremic solutes were determinants for renal function deterioration in patients with moderate CKD.

## Materials and Methods

### Study population

The MASTERPLAN study was a randomized controlled trial (ISRCTN registry number 73187232) that compared a multifaceted treatment approach with the aid of a nurse practitioner to standard care by a nephrologist on cardiovascular outcome in patients with CKD.[21] In total, 788 adult patients with CKD and an estimated creatinine clearance between 20 and 70 ml/min per 1.73m^2^ were included. All patients provided written informed consent, and the study was approved by the medical ethics committee of the University Medical Centre Utrecht. All patients were treated according to the same guidelines. However, the nurse practitioners were specifically trained in motivational interviewing to enhance compliance and promote life-style changes.[21] We performed a nested case-control study. Patients who received a transplant before baseline were excluded from our selection. In order to evaluate the change in uremic solute concentration over time, only patients with at least one year of follow-up were included. Rapid progression was defined according to KDIGO guidelines as a decline of more than 5 ml/min per 1.73m^2^ per year. A single control from the remaining patients was selected at random for each rapid progressor.

### Data collection

Data collection has been described in detail previously.[21] At baseline and annual intervals blood samples were drawn using EDTA tubes and stored at -80°C. Blood samples collected at one year were used to determine follow-up uremic solute concentrations. Information on medical history, physical activity, diet and medication use was obtained using questionnaires. Patients underwent physical examination. Standard laboratory measurements were performed at local laboratories. However, serum creatinine values were calibrated to the central study laboratory.

### Uremic solute measurements

During the study, samples were analyzed in nine separate batches, each batch including both the baseline and follow-up sample for an individual patient to reduce analytic variation. Furthermore, every batch included both rapid and slow progressors to ensure statistical adjustment for potential batch effects was possible. We selected a panel of eleven well known toxins based on previous work. The selected solutes inhibit tubular transport activity,[5] are associated with epithelial phenotypic changes in the renal tubule,[4] or reduce mitochondrial activity in tubular epithelial cells.[18] Uremic solute concentrations were determined with liquid chromatography-tandem mass spectrometry (LC-MS/MS) as described previously.[22] Before chromatography an aliquot of plasma was diluted in H2O (1:1) and deproteinized with perchloric acid (final concentration 3.3% (v/v)). Next, samples were centrifuged at 12,000 x g for 3 min and the clear supernatant was injected into the UPLC-MS/MS system that consisted of an Accela HPLC system coupled to a TSQ Vantage triple quadropole mass spectrometer (Thermo Fischer Scientific, Breda, the Netherlands) equipped with a C18 UPLC column (Acquity UPLC HSS T3 1.8 um; Waters, Milford, MA). The autosampler temperature was set at 8°C and the column temperature at 40°C. The flow rate was 350 μl/min. Eluent solvent A consisted of 10 mM NH4-acetate, solvent B was 5 mM NH4-acetate and 0.1% formic acid and solvent C was 100% methanol. Detection of the components was based on isolation of the deprotonated (negative electrospray; [M-H]-) or protonated molecular ion (positive electrospray; [M+H]+) and subsequent MS/MS fragmentations and a selected reaction monitoring (SRM) were carried out. The UPLC-MS/MS operating conditions and SRM transitions used for parent compounds and ion products were optimized for each component. A calibration curve was used to quantify the solute levels in the samples. Acquired data were processed with Thermo Xcaliber software (Thermo scientific).

### Statistical analyses

Descriptive data are presented as frequencies and proportions, means and standard deviations for normally distributed variables or medians and inter quartiles ranges if the distribution was skewed. X^2^, Wilcoxon ranksum, and t-tests were used to compare baseline covariates between groups. Reference concentrations for healthy controls were obtained from the Human Metabolome database.[23] We compared the mean concentrations in our population to these values. Correlations between baseline covariates and uremic solute levels were obtained from a linear regression and calculated as the square root of R^2^.

The variation in serum levels of uremic solutes was assessed with scatter plots and Bland-Altman plots. The limits of variation were calculated as 1.96 times the standard deviation of the average difference in baseline and follow-up concentration. As the variation in concentration increased with uremic solute level, we took the natural logarithm of the baseline and follow-up concentrations. Back transformation of the difference and its limits allowed us to interpret the variation in uremic solute concentrations as ratios.[24]

Logistic regression was used to estimate odds ratios for rapid progression by uremic solute level. We used the dagitty.net tool to create a causal diagram and identify the minimal variable set to be included into the logistic regression in order to obtain an unbiased estimate of the association between uremic solute concentrations and progression of CKD. The causal diagram can be found in the supplements available online (S1 Fig directed acyclical graph for the analysis of uremic solutes as determinants of rapid progression in CKD).

## Results

### Baseline characteristics

**Fig 1:**
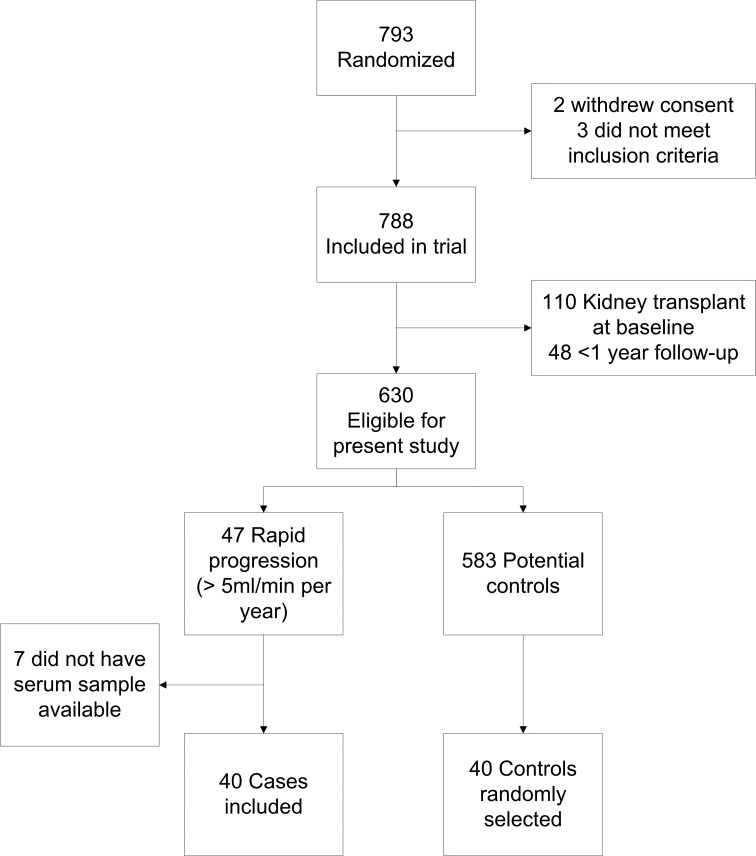
Flow chart for patient inclusion.

Of the 788 patients included in the MASTERPLAN trial, 110 patients had received a kidney transplant prior to baseline and 48 patients had less than one year follow-up and were therefore excluded. In total, 47 of the included patients showed rapid progression. We were unable to retrieve samples for seven of them. Controls for the remaining 40 rapid progressors were randomly selected. Fig 1 shows the flow chart for patient inclusion. Total follow-up duration was 4.4 (inter quartile range 2.9 to 5.0) years. Table 1 shows the baseline characteristics for both groups. The majority of included patients were male and mean age was 63 years in the slow progressors compared to 55 years in the rapid progressors. Mean eGFR was 36 ml/min per 1.73m^2^ for both slow and rapid progressors. In general, patients with rapid progression were younger, were more likely to have congenital kidney disease (86% of whom had cystic kidney disease), had higher proteinuria and uric acid, and a lower serum albumin concentration. Only the difference in baseline serum kynurenic acid concentration, which was higher in rapid progressors, approached statistical significance (Table 2). Levels of uremic solutes in our study sample were generally elevated compared to healthy individuals, with the exception of kynurenic acid, which was lower compared to healthy individuals.

**Table 1:** Baseline characteristics.

Variable	Slow progressors (n = 40)		Rapid progressors (n = 40)		
	mean / median	SD / IQR	mean / median	SD / IQR	p
Men	63%		75%		0.23
Age	64.9	(57.8–70.6)	57.1	(46.2–67.8)	0.07
Caucasians	88%		85%		0.75
Current smoker	23%		28%		0.61
Diabetes	20%		25%		0.59
ADPKD	8%		28%		0.02
Systolic blood pressure [mmHg]	139.6	19.1	139.7	24.1	0.98
Diastolic blood pressure mmHg]	78.5	(69.4–85.5)	82.4	(70.2–86.5)	0.18
eGFR [ml/min per 1.73m2]	35.7	(28.5–44.8)	36.3	(25.7–42.9)	0.65
Proteinuria [g/24h]	0.3	(0.2–0.7)	1.0	(0.2–2.3)	0.01
BMI [kg/m2]	27.5	4.2	26.5	3.5	0.26
Serum cholesterol [mM]	4.6	(4–5.3)	5.0	(4.3–5.9)	0.18
Serum albumin [g/l]	40.8	3.2	39.4	3.3	0.07
Serum calcium [mM]	2.3	0.1	2.3	0.1	0.50
Serum phosphate [mM]	1.0	(0.9–1.1)	1.1	(0.9–1.3)	0.26
Serum bicarbonate [mM]	24.8	3.8	23.9	3.2	0.26
Hb [mM]	8.4	0.9	8.1	1.1	0.21
PTH [pM]	7.2	(4.5–11)	10.2	(7.5–13.6)	0.01
Serum urea [mM]	10.7	(8.2–14.2)	12.6	(9.1–16.8)	0.50
Serum uric acid [mM]	0.4	0.1	0.5	0.1	0.05
Hb1Ac [%]	5.8	(5.5–6.3)	5.9	(5.6–6.6)	0.75
ACEi/ARB	85%		83%		0.76
Any BP lowering drugs	95%		93%		0.64
Anticoagulants	58%		53%		0.65
Vitamin D	13%		23%		0.24
Phosphate binders	5%		3%		0.56
Any lipid lowering drugs	68%		68%		1.00
ESA	5%		5%		1.00
Protein intake (g/d)	70.9	19.0	71.9	24.3	0.84
Fat intake (g/d)	67.3	(56–95.3)	76.1	(59.7–104)	0.49
Cholesterol intake (g/d)	154.9	(117.6–198.2)	168.3	(119.6–215.3)	0.49
Linoleic acid intake	13.2	5.8	13.8	6.1	0.67
Carbohydrate intake (g/d)	231.6	77.8	239.8	94.4	0.68
Dietary fiber intake (g/d)	19.7	(17.4–26.7)	19.6	(15.9–26.2)	0.82
Alcohol intake (g/d)	6.7	(0–17.1)	2.1	(.1–13.6)	0.49
Fluid intake (ml/d)	1691	(1375–2210.7)	1726	(1248.2–2199.6)	0.82

Data are presented as mean ± standard deviation, median (inter quartile range) and proportions. eGFR: estimated GFR, calculated using the abbreviated Modified Diet in Renal Disease equation re-expressed for mass-spectrometry traceable serum creatinine concentration.

**Table 2:** Uremic solute concentrations in CKD patients.

Uremic solute	Reference*	Slow progressors		Rapid progressors		
	Mean (SD or range)	Median	IQR	Median	IQR	p
CMPF [uM]	4.6 (4.2)	5.7	(2.7–14.9)	4.1	(1.7–8.3)	0.17
Hippuric acid [uM]	3.0 (0.0–5.0)	12.8	(5.5–24.5)	12.9	(5.9–24.9)	1.00
Indole acetic acid [uM]	0.05 (0.0–0.118)	3.4	(2.1–5.4)	4.2	(2.6–5.9)	0.17
Indoxyl sulfate [uM]	2.49 (1.36)	16.7	(10.3–21.8)	15.5	(8.8–22.2)	0.65
Kynurenic acid [nM]	230 (10)	122	(94–188)	154	(111–178)	0.07
Kynurenine [uM]	1.6 (0.1)	2.8	(2.4–3.3)	3.0	(2.4–3.6)	0.36
Quinolinic acid [uM]	0.47 (0.047)	0.7	(0.5–1)	0.9	(0.6–1.1)	0.26
p-Cresyl glucuronide [uM]	1.0 (0.7)	0.3	(0.1–0.6)	0.3	(0–0.7)	1.00
p-Cresyl sulfate [uM]	n/a	42	(25–68)	49	(24–82)	0.17
Phenyl sulfate [uM]	n/a	4.9	(2.6–8.2)	5.4	(3.4–11.6)	0.36
Tryptophan [uM]	n/a	43	(33–49)	40	(30–47)	0.36

* Reference values were obtained from the Human Metabolome Database.[23] CMPF: 3-carboxy-4-methyl-5-propyl-2-furanpropionate.

### Association of uremic solute concentrations and baseline covariates

**Fig 2:**
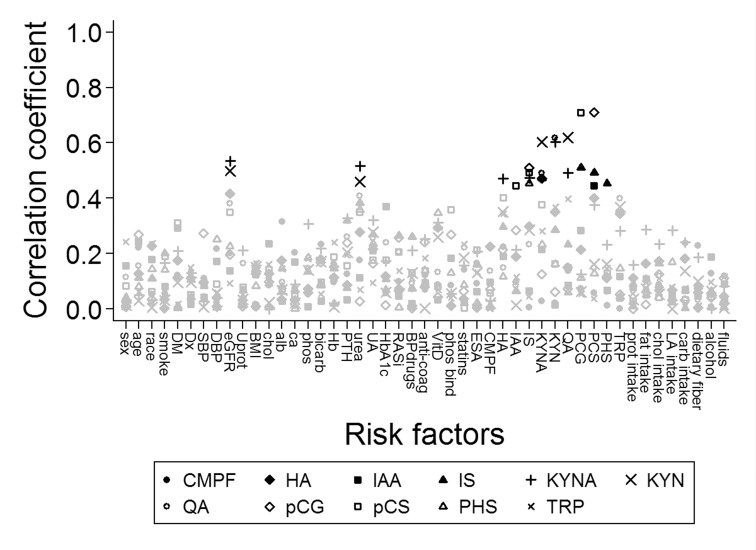
Correlations between uremic solute concentrations and baseline covariates. The square root of the pearson’s R^2^ was taken to achieve positive values. The black symbols mark correlations that were statistically significant at a nominal p<0.05 after Bonferroni correction. DM: diabetes mellitus, Dx: congenital kidney disease, SBP: systolic blood pressure, DBP: diastolic blood pressure, eGFR: estimated GFR, Uprot: proteinuria, BMI: body mass index, chol: serum cholesterol, alb: serum albumin, ca: serum calcium, phos: serum phosphate, PTH: serum parathyroid hormone, UA: serum uric acid, RASi: renin-angiotensin inhibition, BPdrugs: blood pressure lowering drugs, anti-coag: anti-coagulation, VitD: vitamin D use, phos binder: phosphate binder use, statin: lipid lowerin drug use, ESA: erythropoiesis stimulating agent use, CMPF: 3-carboxy-4-methyl-5-propyl-2-furanpropionate, HA: hippuric acid, IAA: indole-3-acetic acid, IS: indoxyl sulfate, KYNA: kynurenic acid, KYN: kynurenine, QA: quinolinic acid, PCG: p-cresyl glucuronide, PCS: p-cresyl sulfate, PHS: phenyl sulfate, TRP: tryptophan, prot intake: dietary protein intake, sat fatty acid: saturated fatty acid intake, MU fatty acid: mono unsaturated fatty acid intake, PU fatty acid: poly unsaturated fatty acid intake, carb intake: carbohydrate intake, % fat: proportion of fat in diet, % protein: proportion protein in diet, % carbs: proportion carbohydrates in diet, % unsat fat: proportion of poly unsaturated fatty acids of total fatty acid intake.

We performed exploratory analyses to determine associations between uremic solute concentration and known risk factors for the development and progression of CKD. Fig 2 shows correlations between uremic solutes and baseline covariates. Remarkably, CMPF concentration only weakly correlated to other uremic solute concentrations. Hippuric acid was statistically significantly correlated with kynurenic acid. Indole-3-acetic acid correlated with p-cresyl sulfate. Indoxyl sulfate associated with p-cresyl sulfate and its glucuronide and phenyl sulfate. Kynurenic acid correlated with kynurenine, quinolinic acid in addition to its correlation with hippuric acid. Kynurenine associated with quinolinic acid as well. Finally, p-cresyl glucuronide and p-cresyl sulfate concentrations correlated too. Additionally, kynurenic acid and kynurenine correlated statistically significantly with eGFR and serum urea concentration. Other base covariates were not strongly correlated to uremic solute concentrations.

### Variability of uremic solute concentrations

**Fig 3:**
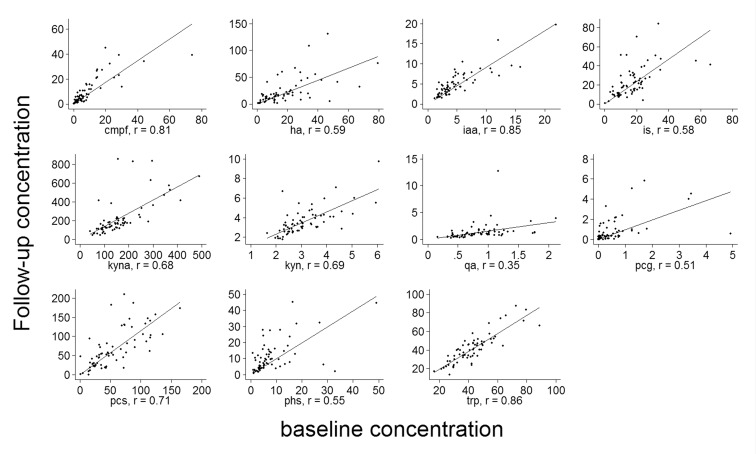
Scatter plots for the concentration in uremic solute concentration at follow-up versus the baseline concentration. The solid line represents the linear fit. Units are in μmol/l for all solutes except for kynurenic acid which was measured in nmol/l. cmpf: 3-carboxy-4-methyl-5-propyl-2-furanpropionate, ha: hippuric acid, iaa: indole-3-acetic acid, is: indoxyl sulfate, kyna: kynurenic acid, kyn: kynurenine, qa: quinolinic acid, pcg: p-cresyl glucuronide, pcs: p-cresyl sulfate, phs: phenyl sulfate, trp: tryptophan.

Fig 3 shows scatter plots for uremic solute concentrations at the follow-up measurement versus baseline concentration. On average, the mean solute levels were slightly higher at follow-up. However, substantial variation was observed, which increased with higher concentrations. We calculated the mean difference and the limits of variation between baseline and follow-up concentrations, which is shown in Table 3. The limits can be interpreted as the bounds in which 95% of all differences between follow-up and baseline concentration will fall. As uremic solute concentrations cannot be negative and their distributions were right tailed, a relative measure of variability was more appropriate. Therefore, we repeated the analysis using log-transformed concentrations. Back transformation gave values that can be interpreted as a ratio between follow-up and baseline concentration. For example, the limits of variation in hippuric acid concentration between baseline and follow-up were 0.24 and 5.6, respectively. Remarkably, tryptophan and its metabolites, indole-3-acetic acid and kynurenine, showed least variation.

**Table 3:** Variability of uremic solute concentrations.

Uremic solute	Mean difference	Limits of variation			Antilog mean difference*	Limits of variation		
CMPF	1.0	-13	-	15	1.13	0.35	-	3.6
HA	4.7	-33	-	43	1.15	0.24	-	5.6
IAA	0.1	-3.8	-	4.0	1.05	0.53	-	2.1
IS	5.5	-20	-	31	1.29	0.43	-	3.9
KYNA	63	-222	-	349	1.23	0.51	-	3.0
KYN	0.5	-1.5	-	2.5	1.13	0.70	-	1.8
QA	0.5	-2.5	-	3.5	1.34	0.46	-	3.9
pCG	0.3	-1.9	-	2.4	1.48	0.11	-	19
pCS	13	-57	-	82	1.14	0.22	-	5.8
PHS	2.8	-14	-	20	1.35	0.28	-	6.6
TRP	-1.4	-17	-	14	0.95	0.64	-	1.4

*The antilog can be interpreted as a ratio. The antilog of the mean difference was calculated by taking the natural logarithms of the baseline and follow-up concentration for each patients. Then the difference between the two was calculated and the mean of these differences for all patients was estimated. Subsequently, the antilog (exponent) of the difference was taken. CMPF: 3-carboxy-4-methyl-5-propyl-2-furanpropionate, HA: hippuric acid, IAA: indole-3-acetic acid, IS: indoxyl sulfate, KYNA: kynurenic acid, KYN: kynurenine, QA: quinolinic acid, PCG: p-cresyl glucuronide, PCS: p-cresyl sulfate, PHS: phenyl sulfate, TRP: tryptophan.

### Association between uremic solutes and progression of CKD

Table 4 shows the odds ratios for rapid progression by baseline serum uremic solute concentrations, crude and adjusted for potential confounders. None of the solutes were statistically significantly associated to rapid progression in the crude analysis. Only indole-3-acetic acid concentration was statistically significantly associated to rapid progression in the adjusted analysis, with an adjusted OR of 2.07 (95% confidence interval 1.07 to 4.02) per incremental standard deviation in concentration. As a sensitivity analysis, the association between uremic solute concentrations and change in eGFR as a continuous variable was also determined, but appeared to be similar as presented in Table 5.

**Table 4:** Odds ratio of rapid progression for a panel of uremic solutes.

Uremic Solute	Crude				Adjusted			
	OR	95% CI			OR	95% CI		
CMPF	0.78	0.48	-	1.29	0.94	0.49	-	1.81
Hippuric acid	1.20	0.76	-	1.90	1.50	0.77	-	2.93
Indole acetic acid	1.25	0.78	-	2.01	2.07	1.07	-	4.02
Indoxyl sulfate	0.84	0.52	-	1.33	0.96	0.54	-	1.70
Kynurenic acid	1.42	0.83	-	2.42	1.39	0.66	-	2.91
Kynurenine	1.24	0.78	-	1.99	1.37	0.76	-	2.48
Quinolinic acid	1.46	0.91	-	2.34	1.21	0.66	-	2.19
p-Cresyl glucuronide	0.90	0.57	-	1.42	0.97	0.51	-	1.84
p-Cresyl sulfate	1.14	0.72	-	1.79	1.38	0.76	-	2.53
Phenyl sulfate	1.12	0.71	-	1.78	1.29	0.72	-	2.32
Tryptophan	0.99	0.63	-	1.57	1.18	0.63	-	2.21

Odds ratios (OR) are presented per incremental SD in uremic solute concentration. The adjusted ORs were obtained from a logistic regression corrected for batch, age, diagnosis (congenital disease present), systolic blood pressure, dietary fiber intake, protein intake, proteinuria and serum albumin concentration at baseline. CMPF: 3-carboy-4-methyl-5-propyl-2-furanpropionate.

**Table 5:** Baseline uremic toxin concentrations as predictors of eGFR decline.

Uremic Toxin	Univariate				Multivariate			
	Change in GFR	95%CI			Change in GFR	95%CI		
CMPF	0.63	-0.23	-	1.48	0.53	-0.34	-	1.40
Hippuric acid	0.02	-0.85	-	0.89	-0.14	-0.94	-	0.66
Indole acetic acid	-0.38	-1.25	-	0.48	-0.95	-1.73	-	-0.18
Indoxyl sulfate	0.49	-0.38	-	1.35	0.24	-0.55	-	1.03
Kynurenic acid	-0.16	-1.03	-	0.71	-0.01	-0.96	-	0.93
Kynurenine	-0.07	-0.94	-	0.80	-0.29	-1.06	-	0.48
Quinolinic acid	-0.42	-1.28	-	0.45	-0.19	-0.96	-	0.59
p-Cresyl glucuronide	0.12	-0.75	-	0.98	0.10	-0.74	-	0.94
p-Cresyl sulfate	-0.29	-1.16	-	0.57	-0.60	-1.40	-	0.20
Phenyl sulfate	-0.14	-1.01	-	0.73	-0.34	-1.12	-	0.45
Tryptophan	-0.09	-0.96	-	0.78	-0.26	-1.05	-	0.53

Change in eGFR is presented per incremental SD in uremic solute concentration. The adjusted changes in eGFR were obtained from a linear regression corrected for batch, age, diagnosis (congenital disease present), systolic blood pressure, dietary fiber intake, protein intake, proteinuria and serum albumin concentration at baseline. CMPF: 3-carboy-4-methyl-5-propyl-2-furanpropionate.

## Discussion

### Main findings

Advanced stages of chronic kidney disease are characterized by the retention and accumulation of uremic solutes, yet it remains unclear whether uremic solutes are a product of CKD or act as culprits in disease progression. The current study demonstrated that serum concentrations of uremic solutes were elevated in patients with CKD compared to healthy persons. Additionally, the results indicate that serum levels of several uremic solutes were generally not associated with a rapidly progressive decline in eGFR over five years of follow-up within a cohort of CKD patients with poor kidney function, possibly due to lack of power as a result of small sample size, substantial between patient variation in solute concentrations and variable solute concentrations within individual patients over time. Indole-3-acetic acid was seemingly associated to rapid progression of CKD. However, the association was only present in the multivariate model, and no other uremic toxins were associated with rapid progression of CKD, including those within the same metabolic pathway, most notably tryptophan. Therefore the association between indole-3-acetic acid and progression may very well be the product of collider bias. Finally, in all multivariate logistic regression models, low serum albumin, high proteinuria and congential cause of kidney disease were associated with rapid progression of CKD (data not shown).

### Relation to other studies

#### Uremic solute concentrations are elevated in CKD stages 3 and 4

By showing that uremic solutes are elevated in patients with moderate CKD, our results corroborate previous work by others and ourselves.[5, 25, 26] Uremic solutes have been hypothesized to contribute to eGFR decline in CKD. Uremic solutes, such as hippuric acid, indole-3-acetic acid, kynurenic acid have been shown to inhibit tubular excretion by competing for organic anion uptake transporters and efflux pumps.[5, 27, 28] Therefore, elevated uremic solute plasma concentrations increase the risk of drug interactions and toxicity. Moreover, several uremic solutes contribute to epithelial-to-mesenchymal transition in the renal tubule.[6–8] However, elevated levels of these uremic solutes were not associated with a more rapid eGFR decline in the present study. Overall, there is little evidence to support a direct effect of individual uremic solutes on progressive eGFR decline in patients with moderate CKD.

#### Uremic solute concentrations vary greatly over time

Our findings indicate that intra-individual uremic solute levels were highly variable. Eloot and colleagues have shown a large variation in uremic solute concentrations between patients, and poor correlation between eGFR and concentrations of several solutes.[29] Importantly, they showed that variability in solute concentrations between patients increased with decreasing eGFR, reflected by increasingly greater standard deviations. In a follow-up article the authors argued that this might mean that, indices of tubular function become more important as renal failure progresses.[30] This view is corroborated by the results of the IDEAL study, in which three quarters of all patients randomized to a late start of hemodialysis, initiated dialysis prior to achieving the target eGFR due to uremic symptoms.[31] GFR, and its decline, may not be the only important outcome parameter in progressive CKD. Another, more recent, study reported variability of uremic solute concentrations as well.[32] However, the authors used correlation coefficients to assess variability, which reflects the presence of a linear association rather than variability.[24] Moreover, correlation depended on the range of measurement. Therefore, substantial variation may still be present despite a strong correlation. In contrast, the limits of variability signified the maximum difference between uremic solute concentration at the first and second measurement for 95% of the observations.[24] Our results show that the variability bands for all solute levels were wide. The variability in uremic solute concentrations was evaluated at two time points, one year apart. Therefore, it is unclear whether variability is the consequence of random, day-to-day variation, or indicative of a trend. However, in all likelihood, the variation in uremic solute concentrations in the present study stems from changes in production and/or absorption of solutes, and not from changes in excretion, as excretory function is unlikely to vary to such a great extent within the individual patient. The variability of uremic solute levels could result in regression dilution bias, and therefore in a false negative result if a relation is truly present. On the other hand, random variation can also result in spurious associations, in particular when sample size is limited, giving rise to false positive results. Either way, any study where uremic solutes were measured at a single point in time is difficult to interpret as a consequence.

#### Uremic solutes and progression of CKD

A recent study in a Chinese population of CKD patients showed that p-cresyl sulfate and indoxyl sulfate were associated to renal progression and mortality.[33] Observed p-cresyl sulfate concentrations were comparable to those found in our study. However, the estimated effect for p-cresyl sulfate was larger in the study by Wu *et al*. In contrast to our study, the authors included patients with a wider GFR range. Moreover, the baseline differences between patients showing progression and those who did not were larger compared to our study. As a consequence, residual confounding may have been present.

In an untargeted metabolic study, Niewczas *et al*. also found that baseline p-cresyl sulfate, but not hippuric acid, was associated with progression to end stage renal disease in patients with early stages of CKD.[32] Remarkably, they showed that in these patients, higher concentrations of tryptophan resulted in lower odds of progression. Likewise, Van der Kloet *et al*. showed that the excretion of tryptophan and its metabolite kynurenic acid were increased in diabetic patients who showed progressive increase in albuminuria.[34] In contrast, tryptophan, nor its metabolites kynurenine and kynurenic acid, associated with progression in our hands. However, compared to the patients in the studies by Niewczas *et al*. and Van der Kloet *et al*., our patients had more advanced CKD. Other metabolic pathways may play a role as CKD progresses,[35] and therefore the effects of some solutes will only become prominent at lower levels of GFR. For example, Wu *et al*. showed that both indoxyl sulfate and p-cresyl sulfate were only associated to renal progression and mortality in patients with GFR <45 ml/min per 1.73m2. This suggests that GFR may be an effect modifier for the risk conveyed by uremic solutes on renal progression. Under the sufficient cause model,[36] this would mean that p-cresyl sulfate and indoxyl sulfate only result in renal progression if kidney damage is already present. However, the findings of Wu *et al*. could not be reproduced in our study.

### Strengths and limitations

A strong point of the present study is the prospective collection of all data and that samples were obtained according to standard operating procedures. As a result, there was little missing data and misclassification of categorical variables was unlikely. Additionally, uremic solutes were measured at two points in time to study possible variation in uremic solute concentration. Assay variation was limited by including samples of individual patients in the same batch and in the multivariate analysis we adjusted for between batch variation.

Nevertheless, the present study has its limitations. First, only eleven solutes out of the more than 150 known solutes were studied. Moreover, only the effects of individual solutes on the rate of progression were considered. Progression of CKD may be the result of several uremic solutes acting together, either due to cumulative load of uremic solutes, or possibly through synergistic effects. Furthermore, a biologically relevant solute may have been missed. Secondly, in addition to extruding uremic solutes, the tubular epithelium removes other potentially toxic compounds and drugs from the circulation.[37] As a result of the increased competition for tubular transport, CKD patients may be at higher risk of drug interactions beyond the apparent risk due to a decreased GFR. This risk of drug interactions may result in more frequent episodes of acute kidney injury. Such episodes may have gone undetected in the current study, as serum creatinine was determined once every three months. Moreover, uremic solutes can induce atherosclerosis.[6, 38, 39] Therefore, hypertension may occur and/or be more pronounced, which in turn leads to a more rapid decrease in eGFR. Our follow-up may have been too short, or the contrast in uremic solute exposure too subtle to study the long-term effects of uremic solutes on hemodynamic changes and subsequent eGFR loss. Third, we have used the abbreviated MDRD equation to estimate GFR. Although the eGFR-MDRD gives an unbiased estimate of measured GFR in patients with CKD stages III to V, any GFR estimated from serum creatinine is fairly insensitive to changes in eGFR over time.[40] This may have resulted in an underestimation of the rate of GFR decline as well as misclassification of rapid progression. Fourth, the relatively low number of patients that were included in the study means that statistical power was limited. The small sample size and high between patient variability of solute concentrations resulted in a small power to detect a statistically significant difference. A rough *post-hoc* power calculation indicated that we had about 17% power to detect a statistically significant effect for a standard deviation increment in logged concentration on the rapid progression of CKD. Low power may result in effect size overestimation, therefore the results present here should be interpreted with caution. Fifth, tubular function and not GFR is expected to be more strongly affected by elevated concentrations of uremic solutes. However, in patients with stages III and IV CKD, progression is largely determined by factors causing nephron loss, such as the primary kidney disease, hypertension and proteinuria. The number of remaining, functional nephrons is reflected most accurately by GFR. In later stages of CKD, when few functional nephrons remain, residual tubular function becomes more relevant.[27] It determines the incidence of uremic symptoms, and therefore the onset and timing of renal replacement therapy. In other words, uremic solutes may not be predictive of change in GFR at early stages of CKD, but may very well predict the onset of end stage renal disease at later stages of CKD. Furthermore, dietary intake of tryptophan and tyrosine containing products are major sources for uremic solute formation. Food frequency questionnaires are known to suffer from underreporting and recall bias, and therefore residual confounding due to misclassification of dietary fiber and protein intake may be present. Finally, uremia has been associated with an altered gut microbiome composition and metabolism, which may include a decrease in beneficial and an increase in potentially pathogenic gut microbiota members, and an increase in the production of uremic toxins.[41, 42]

### Future research

The present study has some major implications for future research. First and foremost, the day-to-day variation in uremic solute concentrations and the determinants thereof remain to be elucidated. Researchers may need to measure the levels of solutes over time, and take detailed questionnaires or journals on diet and food preparation to adequately study variation and its determinants. Secondly, our findings imply that eGFR decline may not be a useful outcome in trials intervening on the level of uremic solutes in patients with established CKD. Perhaps investigators should focus on other clinically relevant outcomes instead, such as the frequency of complications, erythropoiesis stimulating agent use or health-related quality of life. Finally, it remains unclear if solutes have independent, cumulative effects or that synergism may occur.

## Conclusions

Serum uremic solutes concentrations are elevated in patients with CKD compared to healthy individuals. However, the elevated levels of several uremic solutes in patients with CKD were not associated with a more rapid decline in eGFR over five years of follow-up, possibly due to lack of power due to a combination of small sample size, high between patient variability of uremic solute concentration, and high within patient variability over time. The latter complicates the interpretation of studies that measure uremic solutes at a single time point and needs to be elucidated.

## Supporting Information

S1 Fig Directed Acyclical graph for the analysis of uremic solutes as determinants of rapid progression in CKD. The causal diagram show the hypothesized causal mechanism and minimal adjustment set for the multivariate analyses. The open nodes indicate variables included in the adjustment set. The grey nodes are unmeasured confounders. HbA1c is blue, as it is assumed to be a mediator for the effect of uremic solutes.
(TIF)

S1 File data_uremicsolutesinckd.dta. The analysis dataset used for all analyses described in the present study.
(DTA)
