# Supplementary figures and images for: Uremic Solutes in Chronic Kidney Disease and Their Role in Progression
Source: PLoS One. 2016 Dec 29;11(12):e0168117. doi: 10.1371/journal.pone.0168117 (PMC5199014; doi:10.1371/journal.pone.0168117)

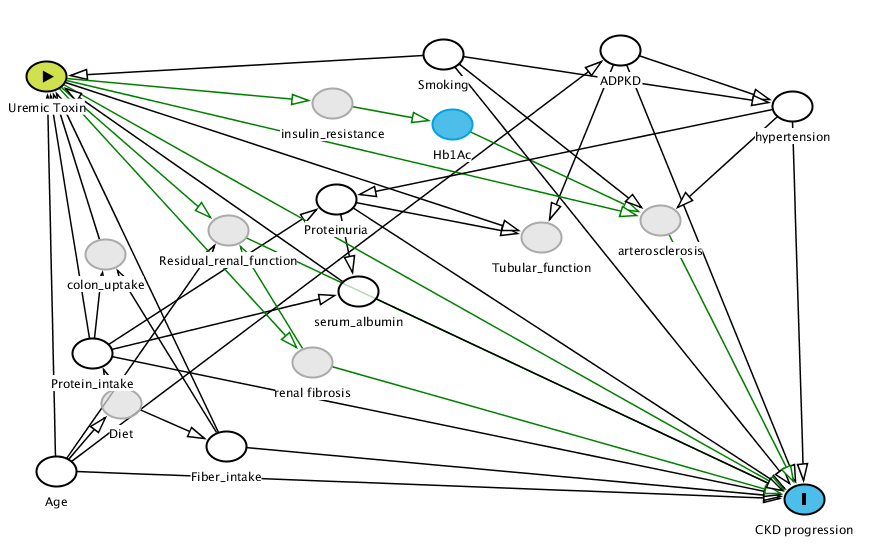

Supplement: S1 Fig — The causal diagram show the hypothesized causal mechanism and minimal adjustment set for the multivariate analyses. The open nodes indicate variables included in the adjustment set. The grey nodes are unmeasured confounders. HbA1c is blue, as it is assumed to be a mediator for the effect of uremic solutes. (TIF) [file pone.0168117.s001.tif]
